# Radiation damage in small-molecule crystallography: fact not fiction

**DOI:** 10.1107/S2052252519006948

**Published:** 2019-06-14

**Authors:** Jeppe Christensen, Peter N. Horton, Charles S. Bury, Joshua L. Dickerson, Helena Taberman, Elspeth F. Garman, Simon J. Coles

**Affiliations:** a Diamond Light Source Ltd, Diamond House, Harwell Science and Innovation Campus, Didcot, Oxfordshire OX11 0DE, UK; b National Crystallography Service, School of Chemistry, Faculty of Engineering and Physical Sciences, University of Southampton, Southampton SO17 1BJ, UK; c Department of Biochemistry, University of Oxford, South Parks Road, Oxford OX1 3QU, UK

**Keywords:** small-molecule crystallography, radiation damage, global damage, specific damage, dose

## Abstract

Traditionally small-molecule crystallographers have not usually observed or recognized significant radiation damage to their samples during diffraction experiments. However, the increased flux densities provided by third-generation synchrotrons have resulted in increasing numbers of observations of this phenomenon. The diversity of types of small-molecule systems means it is not yet possible to propose a general mechanism for their radiation-induced sample decay, however characterization of the effects will permit attempts to understand and mitigate it. Here, systematic experiments are reported on the effects that sample temperature and beam attenuation have on radiation damage progression, allowing qualitative and quantitative assessment of their impact on crystals of a small-molecule test sample. To allow inter-comparison of different measurements, radiation-damage metrics (diffraction-intensity decline, resolution fall-off, scaling *B*-factor increase) are plotted against the absorbed dose. For ease-of-dose calculations, the software developed for protein crystallography, *RADDOSE*-3*D*, has been modified for use in small-molecule crystallography. It is intended that these initial experiments will assist in establishing protocols for small-molecule crystallographers to optimize the diffraction signal from their samples prior to the onset of the deleterious effects of radiation damage.

## Abstract 2

Radiation damage to small-molecule compounds during synchrotron-diffraction data collection, under a range of conditions, is reported, characterized and analysed.

## 1. Introduction   

Although over recent decades radiation damage in macromolecular crystallography (MX) has been widely reported and characterized at 100 K (for reviews see Holton, 2009 ▸; Garman, 2010 ▸; Garman & Weik, 2018 ▸), the same is not true for small-molecule crystallography (SMX), where it has been widely assumed that samples do not suffer from the same radiation-damage effects that have plagued MX since it began. Initially this phenomenon was investigated for the MX case at room temperature (Blake & Phillips, 1962 ▸), but most later investigations were performed under cryogenic conditions, where the crystal is held at around 100 K during data collection and under which around 70 times more data can be obtained compared with room-temperature irradiation (Nave & Garman, 2005 ▸). A substantial body of knowledge pertinent to radiation damage in MX has accumulated over the last 18 years, and some understanding of the physical and chemical processes involved has been established.

Radiation-damage effects can be broadly divided into two classes: global and specific. Global radiation damage occurs in reciprocal space, resulting in the degradation of the crystal and thus in a decrease in the quality of the diffraction data obtained from the crystal. Consequently, global radiation damage is detectable from changes in diffraction-pattern reflection intensities; in particular, it manifests as a gradual fading and ultimate loss of high-resolution reflections. Other observations include expansion of the unit cell, as well as increases in the mosaicity. Conversely, in real space, increased atomic *B*-factors arise and specific damage to particular chemical groups is shown by missing electron density. This occurs in a well established and reproducible order in MX, with metal centres undergoing swift reduction first, then disulfide bonds elongating and breaking, and subsequently aspartate and glutamate residues suffering de­carboxyl­ation, followed by me­thio­nine C—S bonds breaking (Burmeister, 2000 ▸; Ravelli & McSweeney, 2000 ▸; Weik *et al.*, 2000 ▸).

However, for SMX, much less is known about this phenomenon, though two historical studies are worth mentioning here: Abrahams (1973 ▸) and Seiler & Dunitz (1985 ▸). During the 1970s and 1980s SMX diffraction experiments could be very prolonged, and to monitor the stability of both the source and the sample, this led to the best practice of collecting reference reflections at regular time intervals. These studies did show radiation damage taking place over a long period of time, however soon after the introduction of CCD-based area detectors, data collection sped up significantly and the problem seemed to go away. However, with the ever increasing flux densities produced by modern synchrotrons, radiation damage is now again being observed in SMX experiments at 100 K (Morgan *et al.*, 2018 ▸) at which temperature most small-molecule structure determinations are carried out. Small-molecule systems have generally been considered to be more robust than those used in MX, however, in many respects they have much more diversity and accordingly some may be more sensitive to high flux densities than previously thought. We propose that the following range of compounds might be susceptible to radiation damage: highly absorbing metal centres (in coordination complexes, metal-organic framework type materials and inorganic compounds), fragile supramolecular complexes held together by weak intermolecular interactions, hydrates, and solvates. To illustrate the diversity of these systems, a study of the Cambridge Structural Database (CSD; Groom *et al.*, 2016 ▸) for the case of solvates alone (Wiggin & Ward, 2018 ▸; Groom *et al.*, 2016 ▸) shows there to be nine predominant solvate forms (di­chloro­methane, pyridine, benzene, aceto­nitrile, methanol, tetra­hydro­furan, di­methyl­formamide, toluene and chloro­form) but also an equal number of rarer cases observed. Thus it might be expected that there is a range of different mechanisms responsible for radiation-induced decay in small molecules, only some of which are akin to those observed in MX *i.e.* hydrates. So far, reports of these effects in SMX have been anecdotal and, to our knowledge, no systematic studies have yet been published.

Here, the results of comprehensive experiments on the radiation damage suffered by crystals of a test small molecule as a function of temperature (30, 60, 100 and 120 K) and beam attenuation (0.5, 1, 2 and 3% transmission) are reported. To allow for comparison of experimental results between different beamlines and synchrotrons, a commonly accepted metric against which to assess different radiation-damage indicators must be established. In MX, these are plotted against the absorbed dose (energy absorbed per unit mass, J kg^−1^ = Gy, gray), arising because incident photons lose energy in the sample through the photoelectric and Compton effects. The former effect is the major mechanism of energy transfer to the sample, and results in complete absorption of the X-ray with the production of a high-energy photoelectron (incident X-ray energy, typically keV, minus the electron binding energy, typically eV). For an incident 18 keV energy beam, the resulting photoelectron can cause at least 720 further ionizations of atoms in the crystal, thus spreading the damage through the sample (O’Neill *et al.*, 2002 ▸). Since electrons are still mobile at 100 K because of residual thermal energy and quantum mechanical tunnelling (Jones *et al.*, 1987 ▸), they are able to travel through the molecular structure and attach to the sites with the highest electron affinity, hence contributing to the specific damage.

In MX, a commonly used software for estimating the absorbed dose is *RADDOSE*-3*D* (Zeldin *et al.*, 2013*b* ▸; Bury *et al.*, 2018 ▸) and below we report its adaption for use in SMX. The ability to accurately calculate the dose to which a sample has been subjected will enable quantitative and systematic comparisons of radiation-damage investigations. This will provide insight into mechanisms and underpin the development of tools to mitigate radiation-induced decay during SMX experiments. The aim of this article is to demonstrate that radiation damage can readily occur in SMX samples and can have significant effects on the data and models produced. Although here we include the results from only one type of small-molecule sample, we also provide a general methodology to establish and understand the extent of the effects.

## 2. Methods   

The sample used in this study was a nickel(II) complex that crystallizes as a hydrate. The structure was originally elucidated (Carbonell *et al.*, 2013 ▸) as a result of data collection on beamline I19-1 (Nowell *et al.*, 2012 ▸; Allan *et al.*, 2017 ▸) at the Diamond Light Source (DLS). The sample was considered a suitable candidate for this study since a qualitative obser­vation of intensity decay was observed at that time *i.e.* a loss of resolution during the data collection. Additionally, being a hydrate, containing a metal centre and possessing carboxyl­ate functional groups are all also features that are known (see above) to have some bearing on sample decay in the X-ray beam and could be further aspects to investigate. Furthermore, the sample had shown good general stability over time and a regular crystal size and morphology.

### 2.1. Crystal preparation and characteristics   

Crystals of *catena*-bis(μ_2_-glycyl-histidinato-*N*,*N*′,*O*)-nickel(II) heptahydrate (CSD entry FINWUW) were obtained following the process described by Carbonell *et al.* (2013 ▸), where 0.35 ml of 0.3 *M* nickel(II) acetate tetrahydrate solution was mixed into a 2 ml solution of glycine-hystidine (1 m*M*) and the solution was left for 2 h during which time crystals formed. The crystals used for this study had been left in storage in a transparent sample vial for over six months before use and according to light microscopy were perfectly stable under these conditions. The sample was comprised of very pale lilac coloured rod-shaped crystals all of comparable size and shape, so that very similar single crystals could be selected for different diffraction experiments without the need for further manipulation, such as cutting or grinding, in order to undertake a comparable experiment.

**Figure 1:**
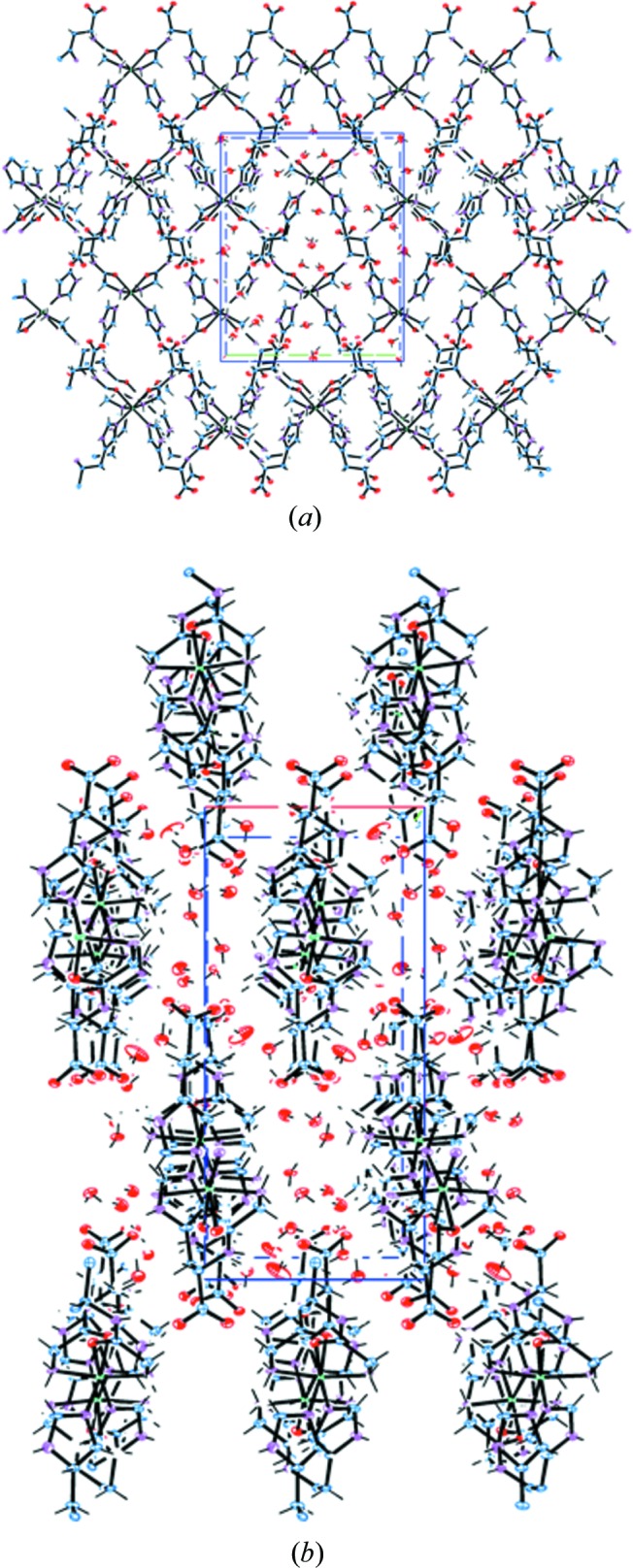
1D polymers extending down the *b* axis (into the page) and (*a*) hydrogen bonding of the hydrates in the channels formed between them. (*b*) A perpendicular view clearly indicating water channels. Carbon is shown in black, nitro­gen is shown in blue, oxygen is shown in red and nickel is shown in green.

A low-dose dataset was collected from one of these crystals on the in-house X-ray facility at Southampton University to create a benchmark for the experiments described below, and the structure submitted to the Cambridge Crystallographic Data Centre (CCDC entry 1901775). There are a number of notable features of the crystal structure. Primarily, it is a hydrate with an unusually high percentage of water for a small-molecule system: seven water molecules in the asymmetric unit, equating to 19.7% of the unit-cell volume, which is 20.6% of the mass [at the time of writing only 1467 structures in the CSD are described as heptahydrates, while it contains a total of *ca*. 116 000 hydrates (Wiggin & Ward, 2018 ▸)]. All of these water molecules are in channels in the structure and therefore none of them directly bind to a nickel centre. The structure, illustrated in Fig. 1 ▸, comprises polymeric 1D tapes extending along the *b* unit-cell axis where di-peptides are linked through the nickel ion. The water molecules lie in channels formed between each tape, with all being involved in hydrogen-bonding interactions.

### 2.2. Beamline calibration   

All experiments presented herein were conducted on beamline I19-1 (Nowell *et al.*, 2012 ▸; Allan *et al.*, 2017 ▸) at the DLS. This is a dedicated small-molecule beamline with a three-circle fixed chi goniometer equipped with a PILATUS 2M PIXEL detector. The beam is conditioned using a double crystal Si-111 monochromator and then focused by two bimorph mirrors. Upstream of the sample, the beam is collimated using a 100 µm platinum:iridium (95:5)% aperture.

The collimated flux of the 18 keV energy X-ray beam was measured with a 500 µm thick calibrated silicon PIN diode, using the protocol established by Owen *et al.* (2009 ▸). Briefly, a Keithley picoammeter was connected to the photodiode (Canberra, model number PD300-500CB) to record the current induced at different beam transmissions by changing the thickness of the aluminium beam attenuators. These currents were then converted into photon fluxes using the known diode-calibration formula. The X-ray beam profile was determined using the existing beamline fluorescent YAG screen (see Figs. S1 to S3 in the Supporting information), resulting in a photon count per 2D pixel in both the horizontal and vertical directions. The pixels were converted to micrometres (the micrometres to pixels ratio being 285:1024) to find the horizontal and vertical FWHM values for the beam, which was approximately Gaussian shaped. In the horizontal plane the FWHM is 150 µm and in the vertical plane the FWHM is 100 µm. This is subsequently modified by a circular 100 µm aperture just before the sample.

Tables S1 to S5 in the Supporting information detail the flux-density calibration data collected over a number of separate visits to the beamline and the data are visualized in Fig. S4. The unattenuated flux was ~8 × 10^11^ photons s^−1^. When comparing the flux-density values for measurements made at 100% transmission at different times, it is clear from the significant variation that beam-flux-density calibration for each session is necessary in order to obtain reliable flux-density values for calculating the absorbed dose.

### 2.3. Dose calculations   

For this study, *RADDOSE*-3*D* (Zeldin *et al.*, 2013*b* ▸; Bury *et al.*, 2018 ▸; http://www.raddo.se), which computes spatially and temporally resolved dose fields in crystals undergoing X-ray irradiation, was adapted for use with SMX.

Firstly, since the beam from I19 at the DLS is defined by a circular collimator rather than the horizontal and vertical slits usually used on MX beamlines, *RADDOSE*-3*D* had to be modified to accommodate this characteristic feature. Circular collimation can now be defined for all beam profiles (*i.e.* top hat, Gaussian and experimental 2D intensity map).

Secondly, the contents of the unit cell need to be defined. When used for MX, *RADDOSE*-3*D* users enter the number of amino-acid residues per monomer and the number of monomers of their protein in the unit cell, as well as the number of heavy atoms per protein monomer. *RADDOSE*-3*D* input also requires a user-provided value for solvent content and the concentration of heavy solvent atoms as millimolar quantities. If the solvent content is not entered, the space in the unit cell not occupied by protein is estimated, and then filled with water. This approach cannot be applied to small molecules for a number of reasons. (*a*) The synthesis reaction to produce the small molecule will often be conducted in the absence of water. (*b*) There is an energetic driving force for a molecule to associate with a reaction or crystallization solvent, forming a solvate complex. (*c*) The conformational demands of large or complex compounds can overcome the drive for close packing, resulting in small spaces between adjacent molecules. Different molecules will have different packing efficiency. (*d*) The unit cell can have an empty void space larger than the volume occupied by a single water molecule *e.g.* in the case of porous frameworks or cavity space in large macrocyclic rings. Finally, (*e*) not all void spaces are solvent accessible (van der Sluis & Spek, 1990 ▸), such as, for example, the centre of a fullerene.

Thus, for SMX cases the empty space cannot be automatically filled with water. However, the entire atomic composition of the unit cell is usually known in advance of the SMX experiment. In the case of calculating the absorbed dose when determining the structure of an unknown sample, information from the synthesis reaction and/or complementary data, *e.g.* from elemental analysis, mass spectrometry or NMR on the product, enable good estimation of the unit-cell contents. If the unit cell is known, then application of the ‘18 Å^3^ per non-hydrogen atom rule’ (Kempster & Lipson, 1972 ▸) combined with the synthesis data above will permit a simple calculation to determine whether the unit cell is full or not. A discrepancy between the calculated occupancy and the volume of the unit cell would enable an estimation of the number of atoms occupying the unaccounted space, which combined with synthesis information would provide a very strong indication of the nature and number of solvent molecules. Once the unit cell has been filled, *RADDOSE*-3*D* calculates the dose in exactly the same way as for MX. Alternatively, when a known structure is being investigated, the unit-cell composition can be manually entered or automatically extracted from a CIF in the same way that *RADDOSE*-3*D* currently allows the MX user to calculate the dose from a locally saved PDB file.

The SMX-adapted version of *RADDOSE*-3*D* is available at: https://github.com/GarmanGroup/RADDOSE-3D.

### 2.4. Data collection   

In order to comparatively study radiation damage and to provide meaningful results, a crystal sample was selected that exhibits moderate susceptibility to radiation-induced decay (see Section 2.1). This enables decay effects to be quantified and permits several consecutive datasets to be collected in order to monitor various metrics as a function of dose, compare between conditions and identify specific structural effects without the datasets becoming overly compromised because of decay effects.

The beamline configuration was chosen to be as close to the preferred routine service crystallography arrangement as possible, from which systematic alterations can be made, with a wavelength of 0.6889 Å (17.7997 keV, Zr absorption edge), 1% attenuator transmission and a sample temperature of 100 K. The data-collection strategy employed was to perform three 170° ω scans with phi at 0, 120 and 240°, respectively, which was repeated several times in order to monitor diffraction over a significant dose range. The authors have decided to make the raw data available via https://zenodo.org/ so that those with interest in radiation damage can perform their own studies and/or use the data to test software or as training examples. Further information can be found in the Supporting information, where a specific link to each raw data collection is provided in Table S6.

While MX and SMX use essentially the same methodology, the actual experiments and desired data differ somewhat. Besides the specific characteristics of the composition of the crystals themselves, (*e.g.* the nature/arrangement of water, other solvents of crystallization, the extent of the organic part of a molecule and the presence of heavy elements) there are differing experimental parameters (*e.g.* related to the energy of the X-ray radiation used, expected resolution limit of the data and the total irradiation time to be considered when comparing decay phenomena between MX and SMX). Table 1 ▸ details the primary differences expected to have a significant impact on a sample, and subsequent data, that is prone to decay in the X-ray beam.

### 2.5. Experiment design   

In order to probe the effect of potentially influential parameters on the extent and rate of radiation damage, a related series of experiments were conducted. Table 2 ▸ indicates the parameters investigated and the relationships between the different experiments. The dose was calculated with *RADDOSE*-3*D* using the beam-calibration information (Tables S1 to S5), the incident wavelength and the crystal contents and size.

## 3. Results   

### 3.1. Baseline measurement   

From a crystal of dimensions 55 × 10 × 10 µm, 18 scans (the three ω scans indicated in Section 2.4, repeated six times) were collected in order to fully sample the diffraction lifetime of the crystal (experiment 1). It was possible to process 16 scans, after which the crystal diffraction had very significantly degraded and the processing software failed. The data were integrated using the software package *XIA2* (Winter, 2010 ▸). All scans were integrated to the resolution determined by the experimental geometry (0.59 Å), rather than being truncated at the resolution limit suggested by the software. Based on the beamline calibration and the size of the crystal, the dose for one ω scan was calculated as 0.63 MGy [average diffraction weighted dose (DWD); Zeldin *et al.*, 2013*a* ▸] using the modified *RADDOSE*-3*D* software (see Section 2.3). This was achieved by inserting attenuators to reduce the flux of the incident beam to 1% of that available. It was then possible to assess a range of data-quality metrics as a function of dose, and therefore compare experiments with varying parameters.

### 3.2. Effects on the resulting data of increasing dose   

**Figure 2:**
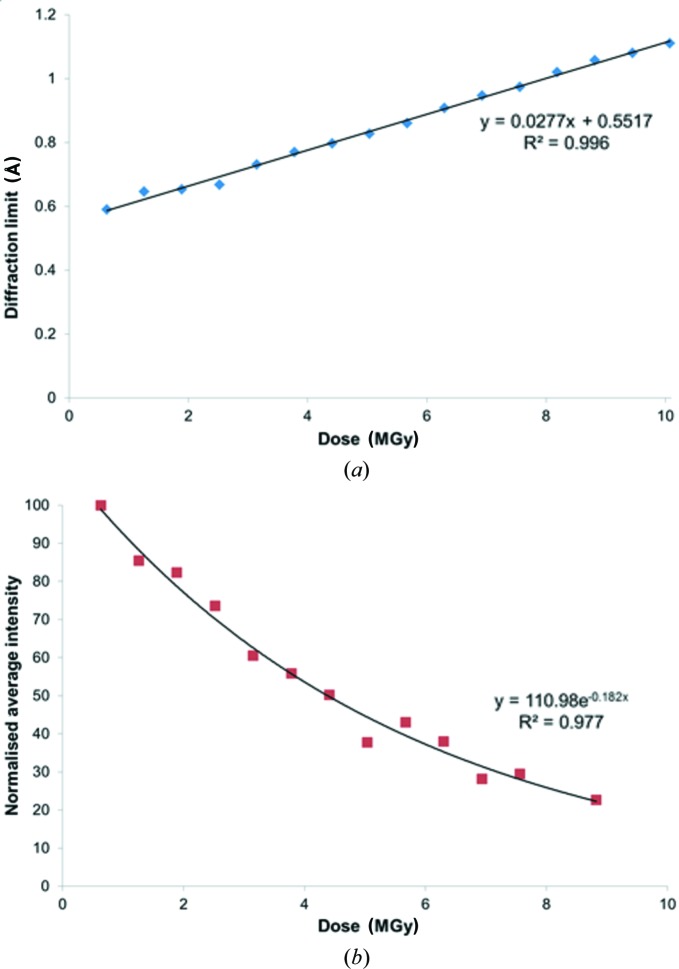
Results from experiment 1. The effect of sample decay at 100 K on (*a*) diffraction limit and (*b*) normalized average intensity as a function of dose.

As the order of a crystal structure degrades, the quality of the resulting diffraction pattern will also decrease. Two well established measures of this are the maximum resolution (as defined below) and the normalized summed diffracted intensity of a dataset as a function of absorbed dose, as illustrated in Fig. 2 ▸. Other parameters that are typically observed to change with radiation damage in MX are the unit-cell dimensions. When these are plotted against dose (see Fig. S5), it can be seen that the change in volume is about 2% for a 10 MGy dose. This expansion is very comparable with that observed in MX (Garman, 2010 ▸) but is seen to be highly variable even between crystals of the same protein (Murray & Garman, 2002 ▸) and is thus not generally used as a metric to assess the level of damage.

These parameters all show the expected behaviour indicative of progressive radiation damage increasing in proportion to the absorbed dose. The diffraction limit is calculated based on the point at which CC_1/2_ (Karplus & Diederichs, 2012 ▸) falls below a threshold (the default value used in the *XIA2* software for this work is 0.3). CC_1/2_ is a correlation coefficient for anomalous differences and the equivalent mean intensity, 〈*I*〉, between two halves of a randomly split dataset. The CC_1/2_ data plotted against resolution for increasing dose is shown in Fig. S5. It can be seen that there is a significant reduction in the diffraction limit as a function of the absorbed dose which is approximately linear (*R* ^2^ = 0.996) and falls from 0.6 to 1.1 Å during the course of the experiment. The average intensity, which is normalized to 100 for comparison between studies (see *infra*), also decreases markedly as the dose increases and follows an expected exponential behaviour (*R* ^2^ = 0.977). The ‘half dose’, or dose to half intensity, *D*_1/2_, is just 4.4 MGy (average DWD), which is due to the very high resolution diffraction and is significantly less than the usual 10–20 MGy observed for protein crystals [*e.g.* 17 MGy to 1.6 Å resolution (Teng & Moffat, 2002 ▸), and between 12.5 and 12.9 MGy maximum dose for 1.8 Å resolution (De la Mora *et al.*, 2011 ▸) for lysozyme at 100 K]. It is also significantly lower than the 30 MGy *D*_0.7_ dose limit at 100 K for 2.2 Å resolution data used as a yardstick in MX, and beyond which biological information may be significantly compromised by specific damage (Owen *et al.*, 2006 ▸). However, as noted in that study, the *D*_1/2_ depends on the resolution of the reflections being included in the intensity-loss calculation, since the diffraction fades fastest in the highest resolution shells. From a meta-analysis of data available at the time, Howells *et al.* (2009 ▸) concluded that the resolution would fall by 1 Å for every 10 MGy of absorbed dose. The crystal studied here initially diffracted to 0.6 Å, but after 4.4 MGy, this fell by 0.5 Å to 1.1 Å, consistent with the Howells prediction.

Note that since the dose absorbed by a Gaussian-shaped beam results in a dose profile in the crystal, different publications quote different dose metrics, ranging through average DWD, through average dose and up to maximum dose. Throughout this article we use average DWD (Zeldin *et al.*, 2013*a* ▸), which in these cases (large beam on a smaller crystal so that the beam intensity is fairly uniform across the crystal) is approximately half the maximum dose (*i.e.* the dose at the most damaged voxel of the crystal).

### 3.3. Effect on atomic model   

**Figure 3:**
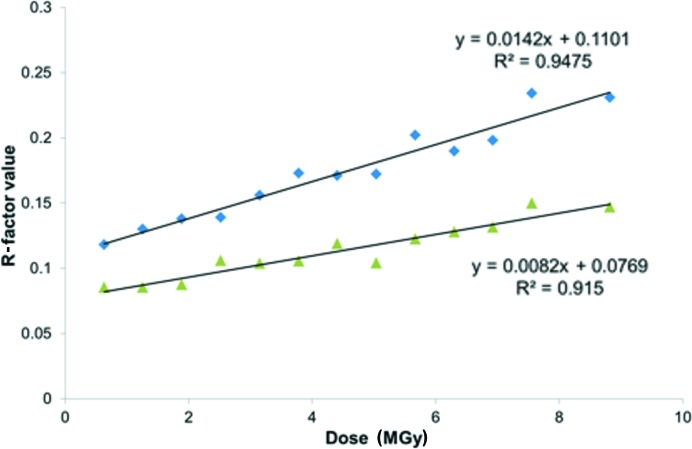
The change in *R*_merge_ (diamonds) and traditional refinement atomic *R*-factor (triangles) values as a function of absorbed dose, where 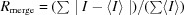 and 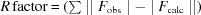 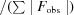.

If data quality is compromised, then the agreement when equivalent reflections are merged will decrease, and thus the merging *R* values will rise. In addition, the atomic *R* value (the traditional SMX *R* factor is used here), which gives a measure of the agreement between the data and the model, will increase. The effects of radiation-induced decay on some agreement factors for merging equivalent reflections and on the agreement between the observed and calculated structure factors, *F*_o_ and *F*_c_, are plotted in Fig. 3 ▸.

The *R*_merge_ and *R*-factor values rise with increasing dose as expected *i.e.* while *R*_merge_ doubles during the course of the experiment, the *R* factor increases by about 50%. Furthermore, by comparing the data in Figs. 2 ▸ and 3 ▸, it can be concluded that relatively speaking the diffraction limit and average intensity are more sensitive metrics compared with *R*_merge_ and *R*-factor indicators.

**Figure 4:**
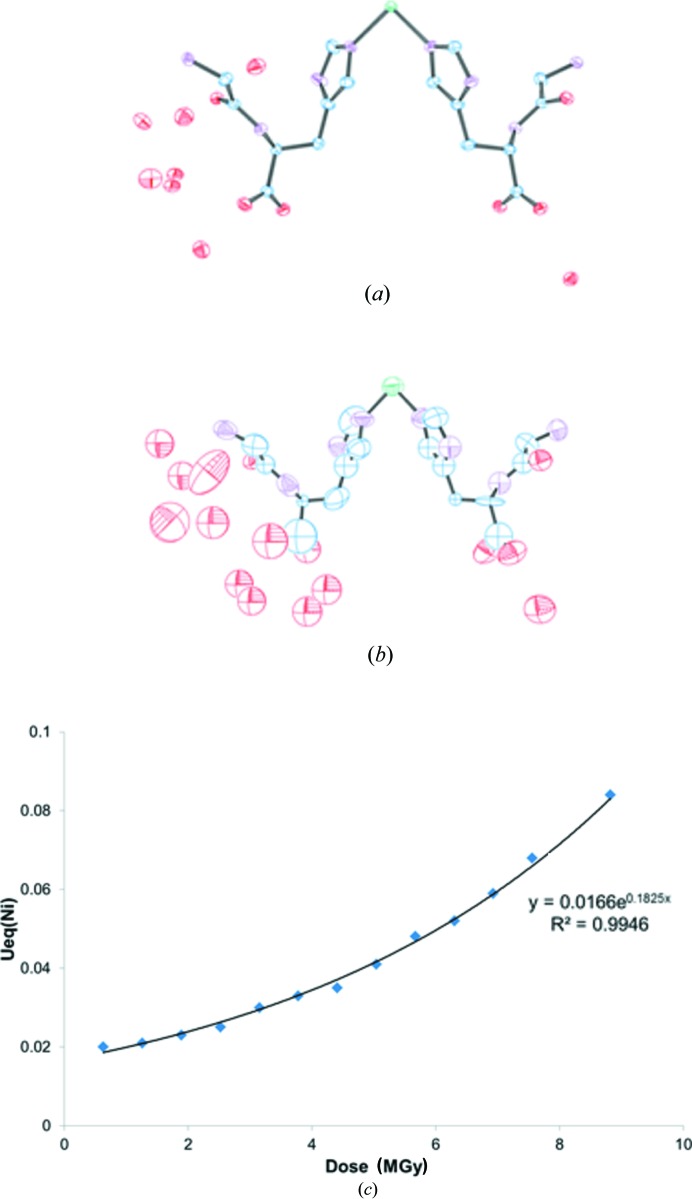
Thermal ellipsoid plots based on (*a*) the first *i.e.* 0.63 MGy and (*b*) the 14th *i.e.* 8.82 MGy scans. Carbon is shown in blue, nitro­gen is shown in purple, oxygen is shown in red and nickel is shown in green. (*c*) The graph shows the development of *U*_eq_ for the nickel site as a function of dose.

The fact that the *R* factor is a less sensitive indicator of radiation-damage progression may reflect the ability of the model to absorb or hide the effects of radiation damage. Overall, the thermal ellipsoids increase as a function of dose, as illustrated by the growth of the equivalent isotropic atomic displacement parameter (*U*_eq_) for the nickel site which exhibits an exponential relationship (Fig. 4 ▸). The details of the structural models change dramatically with increasing dose. At the lowest dose the structure contains seven solvent water-molecule sites in the asymmetric unit, one of which is split into two positions with partial occupancy. After the fifth scan (3.15 MGy) a second water site becomes split and after the eighth scan (5.04 MGy) this increases to three. Finally, after the 11th scan (6.93 MGy), the carb­oxy­lic acid group surrounded by the split water sites also exhibits significant disorder, to the point where a satisfactory disorder model could not be found as the data quality had deteriorated too much.

**Figure 5:**
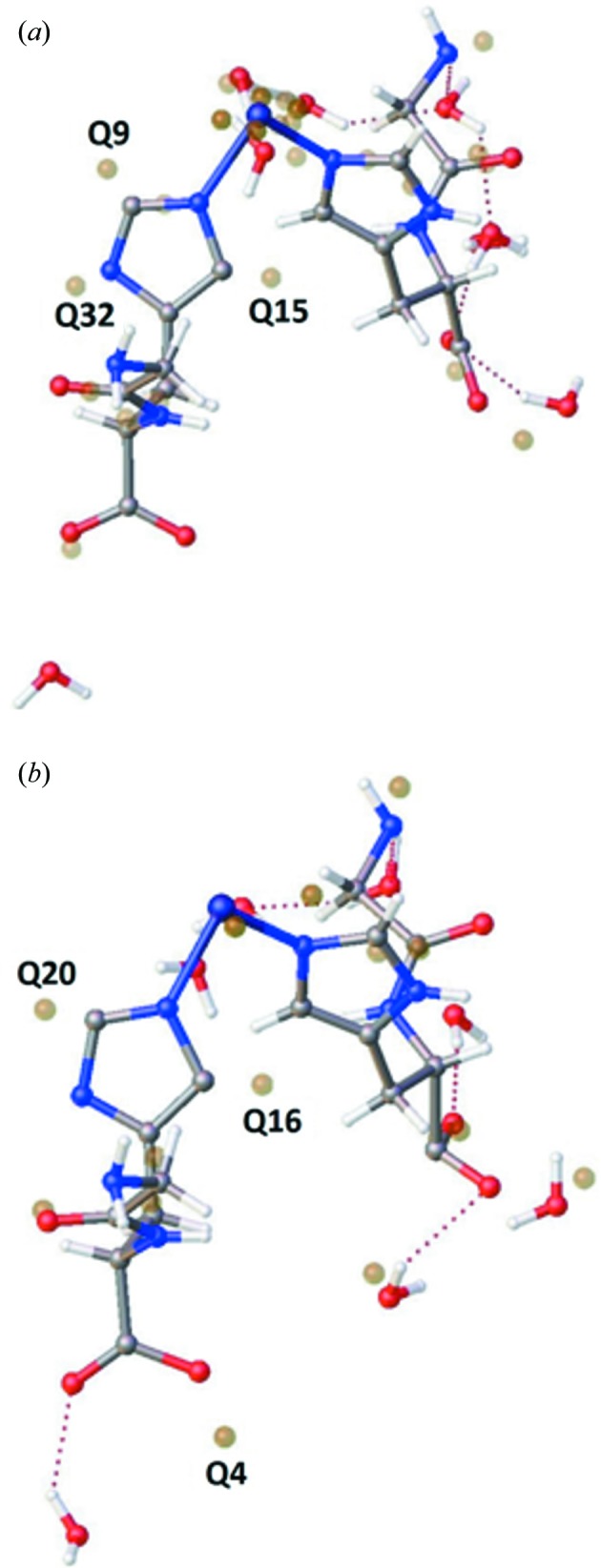
Residual density plots based on data from (*a*) the first scan, *i.e.* 0.63 MGy, and from (*b*) the 12th scan, *i.e.* 7.56 MGy. Carbon is shown in grey, nitro­gen is shown in blue, oxygen is shown in red, hydrogen is shown in white and nickel is shown in dark blue/purple. Residual peaks are coloured brown and the numbered Q labels refer to a ranked peak height. Values for residuals in hydrogen positions are 0.48–0.63 e Å^3^ and 0.31–0.40 e Å^3^ for structures shown in (*a*) and (*b*), respectively.

Reduction in data quality will also cause difficulties in interpreting the model and this point is illustrated by our study. Given that the ligand in this structure is a dipeptide, in its unbound state it may exist as a Zwitterion, and when coordinated the presence or absence of a proton on amino and carboxyl­ate groups confirms the metal oxidation state and overall charge. Data taken from the first scan [0.63 MGy dose, Fig. 5 ▸(*a*)] produce a model where residual peaks corresponding to hydrogen atoms are observed in all positions, but not for the carboxyl­ate moiety. Conversely, data from the 12th scan [7.56 MGy dose, Fig. 5 ▸(*b*)] clearly exhibit a residual peak corresponding to a carboxyl­ate hydrogen atom. This point is amplified by investigation of the bond lengths in this functional group. The 0.63 MGy structure has C1–O1 = 1.252 (8) Å and C1–O2 = 1.272 (7) Å which are essentially equal, however the 7.56 MGy structure has C1–O1 = 1.44 (2) Å and C1–O2 = 1.24 (3) Å which are significantly different. This is indicative of the 0.63 MGy structure possessing a carboxyl­ate group, which as a result of exposure to radiation is transformed into a carb­oxy­lic acid in the 7.56 MGy structure.

### 3.4. Consistency and appropriate metrics   

**Figure 6:**
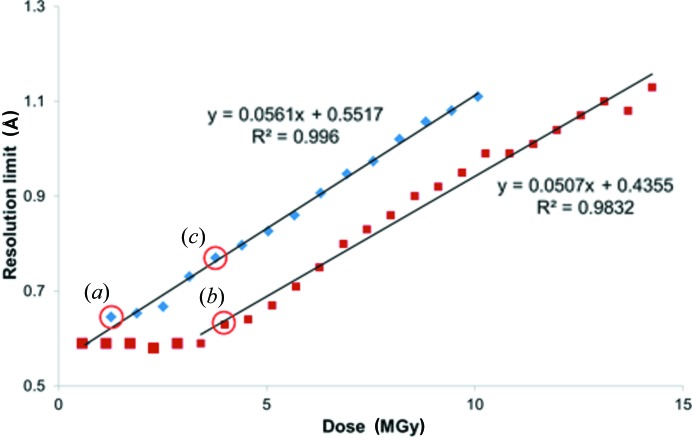
The variation in suggested resolution limit (calculated from CC_1/2_) as a function of dose for experiment 1 (diamonds) and experiment 4 (squares). The two experiments were performed at the same beamline settings and the experimental resolution limit was 0.59 Å. Circles (*a*), (*b*) and (*c*) indicate datasets with similar resolution [(*a*) and (*b*)] or similar dose [(*b*) and (*c*)] (see discussion below).

To confirm that it is valid to compare different experiments using dose as the common metric, a new experiment was performed during a second visit, but under similar conditions (experiment 4). On the second occasion the crystal was 25 × 10 × 5 µm in size and the calculated dose for one scan was 0.57 MGy. This enables a two-crystal comparison, which is presented in Fig. 6 ▸.

**Figure 7:**
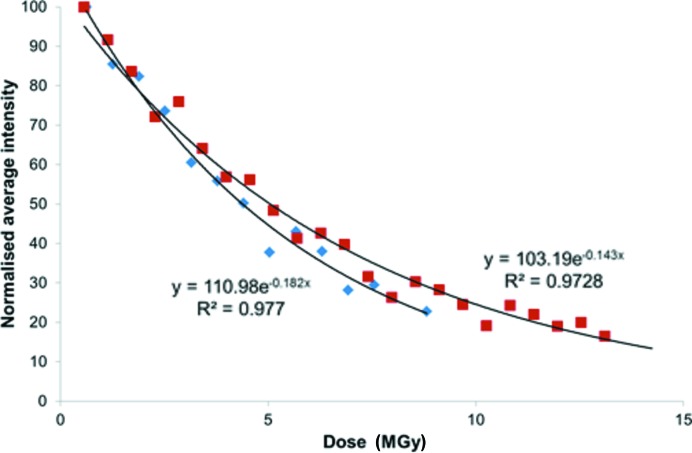
Average integrated intensity (normalized to 100) for each scan for experiment 1 (diamonds) and experiment 4 (squares).

The two curves in Fig. 6 ▸ are shifted with respect to each other, which is due to an expected difference in initial diffraction quality because of the individual nature of the two crystals. When decay effects begin to manifest themselves, a reduction in resolution then results for both samples. However, what is noticeable is that the two curves are parallel to one another, indicating that once decay initiates it proceeds via exactly the same mechanism and at the same rate. The conclusion therefore is that calculating dose is a valid approach and is reproducible across samples; however, the beam flux and profile must be quantified *e.g.* so that a calibration can be made for quantitative studies which can then be compared. However, the results presented here highlight the disadvantage of using resolution as a metric without considering the absorbed dose. Taking the same two sample datasets, an analysis of the normalized integrated intensities (see Fig. 7 ▸) as a function of dose, *i.e.* comparing subsequent scans, illustrates that the two samples decay at the same rate.

**Figure 8:**
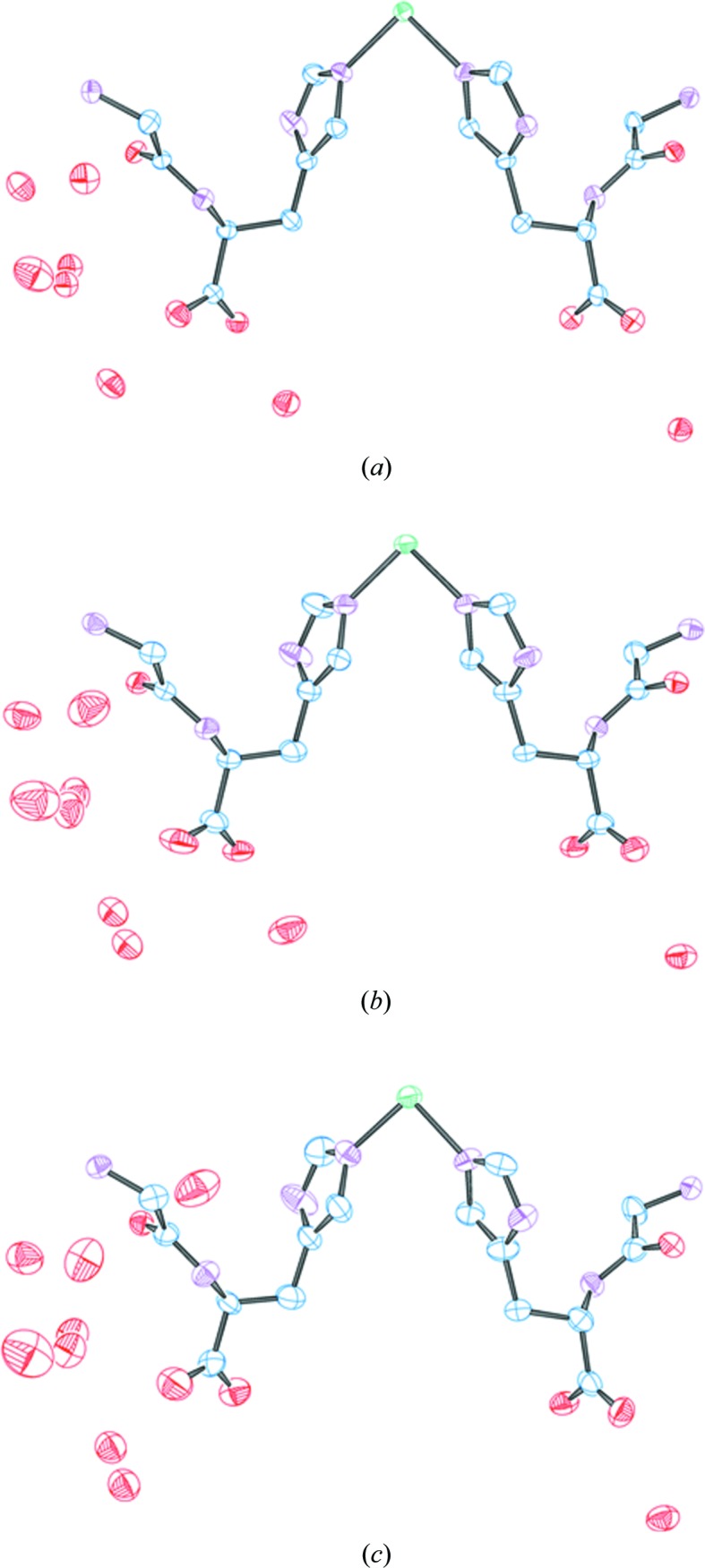
Comparison of structural models for the three data points [(*a*), (*b*) and (*c*)] indicated in Table 3 ▸. Carbon is shown in blue, nitro­gen is shown in purple, oxygen is shown in red and nickel is shown in green.

The results from the same two datasets shown can be used as a comparison of the structure quality from (*a*) two datasets at approximately the same resolution (0.645 and 0.63 Å) but having absorbed a different dose and (*b*) two datasets with approximately equivalent (3.78 and 3.99 MGy) dose but differing resolution. Some data-quality indicators for this comparison are given in Table 3 ▸, and thermal ellipsoid plots for the derived models are presented in Fig. 8 ▸. It can be seen that while there are more marked differences in the metrics the dose-equivalent data provide similar results when comparing the thermal ellipsoid plots. Conversely, the differences in quality metrics for resolution-equivalent data are less marked, yet the thermal ellipsoids are considerably different. From this we can conclude that an increase in dose has a pronounced effect on the model but does not so readily manifest itself in the quality metrics.

### 3.5. Parameters influencing radiation-damage decay rates   

Historically, radiation damage in SMX has been unanticipated and thus there has been no planning on how to mitigate its effects and the problem is not addressed until it is observed during the course of an experiment. Generally, attempts are made to process the data in a manner that will account for the phenomenon *e.g.* in scaling, and in providing caveats as to why model quality is compromised. In the smaller number of cases where this effect really has to be addressed, the perceived (but untested) wisdom has been to adopt one of a range of mitigation strategies.

These approaches generally involve either lowering the data-collection temperature or attenuating the incident X-ray beam and thus reducing the dose rate. However, in the latter case it is often necessary to increase the exposure time, with the result being the same total dose. An alternative approach, generally used at synchrotron sources, is to decrease the exposure time, sometimes in combination with increasing the dose rate. Finally, also at synchrotron sources, another approach has been to change the X-ray energy (wavelength) to avoid excitation effects that may occur for certain elements at absorption edges.

It should be noted here that there are a number of (assumed or proposed) mechanisms for radiation-induced decay in small molecules and the different mitigation strategies that have traditionally been employed on an *ad hoc* basis will have different effects on this process, potentially ranging from nothing to having significant impact. We now outline a series of comparison studies that systematically investigate these different mitigation approaches, with the aim of quantifying their effects.

To investigate the issue of dose rate, fresh crystals were exposed to 2% (experiment 2) and 0.5% (experiment 3) of the incident-beam intensity. When dose is calculated and used to plot the resolution limit (see Fig. S7) the same linear behaviour as experiments 1 and 4 is observed, from which we can conclude that radiation-damage rate is independent of dose rate at the flux densities and sample temperature used here (1.12 × 10^12^ photons s^−1^ mm^2^ and 100 K, respectively). The independence of this relationship has been established in MX up to flux densities of 10^15^ photons s^−1^ mm^2^ (Sliz *et al.*, 2003 ▸).

The effect of sample temperature on the decay rate was investigated by performing experiments at 120, 100, 60 and 30 K (experiments 8, 1, 7 and 6, respectively). The resolution limit as a function of dose for each temperature is plotted in Fig. S8. An immediate observation is that temperature does play a role, but going from 100 K to 30 K only changes the damage rate (as measured by the inclination of the linear section) by approximately a factor of two. This is important as many other parameters such as crystal size, beam intensity, and experimental strategy, can cause a factor of two change in the diffraction output. Put simply ‘a factor of two roughly corresponds to the decision thresholds that must be faced in data collection strategy’ (Holton, 2009 ▸).

We also tested the effect of photon energy by tuning to a different wavelength (experiment 5). As can be seen from the graph in Fig. S9, the linear behaviour is independent of photon energy. It is important to note that this may well not be the case if the beam energy is tuned close to an absorption edge of an element contained in the sample.

## 4. Discussion and future outlook   

The goal of this work was to systematically illustrate the phenomenon of radiation-induced damage in SMX and develop a process to quantify it so that approaches may be developed in the future to mitigate it. From the results of this work it is clear that damage occurs under all the conditions explored and that the extent of this damage can be affected by varying the data-collection parameters. However, temperature is the only variable examined that has an appreciable influence.

These results confirm that for a given 〈*I*/σ(*I*)〉 requirement for an SMX data collection, a particular absorbed dose will result. This is the fundamental determining factor regarding the extent to which radiation decay will occur, which cannot be altered by changing data-collection parameters. It is also clear that the solvent in the SMX samples studied here can become disordered quite readily as a function of dose, and that for longer, *i.e.* higher dose, data collections, molecular geometry can become less reliable. In summary, loss of data quality is a factor that should be considered when conducting the experiment and is very likely to occur. Even for relatively low doses, a reduction in data quality can begin to effect subtle factors such as hydrogen-atom location. Significant observations in this study are that there can be localized damage, such as specific structural changes, or global damage, such as loss in resolution or a general increase in thermal parameter values. Reduction in diffraction limit and decrease in average intensity is consistent with increasing dose. However, the model also decreases in quality, not just in terms of degree of goodness of fit, but also in its physical relevance *e.g.* splitting of sites. In fact, the effect on the physical meaningfulness of the structure is impacted more than are the quality statistics.

Accordingly, the findings of this study pose the fundamental question, does the majority of disorder in SMX models derived from synchrotron data arise from radiation damage? This leads to a hypothesis that this disorder would therefore not be present in a low-dose crystal structure.

Understanding dose rate is therefore a key factor. The obvious approaches that would seem intuitive to a chemical crystallographer, *i.e.* faster data collection or reduction in incident-beam intensity, have no effect (for this case at least) if the dose remains the same, since the basic physics involved in the X-ray absorption processes is unaltered at the flux den­sities used here. This means it is not possible to outrun this decay process and other approaches are required. Lowering temperature is potentially one approach, but decay behaviour is not linear and given the limited availability and high cost of helium, it is not a sustainable approach. Although there are contradictory experimental results in MX on this issue, a similar conclusion has been reached. Decreasing the data-collection temperature further to 40 K yields only a very small decrease in specific and global radiation damage compared with at 100 K (Meents *et al.*, 2010 ▸, 2007 ▸; Chinte *et al.*, 2007 ▸). An exception to this observation is a reported large effect on the rate of metal reduction in metallo-proteins which was reduced 30-fold when collecting data at 40 K instead of 110 K (Corbett *et al.*, 2007 ▸). Thus helium cooling has not replaced nitro­gen cooling in standard cryocrystallographic MX experiments.

The relationship between dose rate and the extent and nature of the damage is quantifiable and it will be possible to use this to design data-collection strategies to limit decay effects. In time, enough understanding of the nature and chemistry of different systems will be accrued and enable a more predictive approach to minimizing radiation-damage effects in SMX.

This work has demonstrated that sample decay as a result of absorbed radiation dose can be a significant factor in SMX. The question that now needs to be addressed is whether this is a matter for concern or not, can it be ignored or should it be addressed? Ultimately the answer to this question depends on the goals of the experiment: the desired outcome for a routine structural characterization is often to confirm composition and conformation and this can generally be achieved. However, for more subtle, involved, and lengthy investigations such as charge-density analysis, phase-transition monitoring, gas adsorption and photocrystallography, the decay effects will be highly significant, and are likely to impact the quality of the results and we encourage the community to investigate the propensity for radiation-induced crystal decay in these areas.

The coming decade will see fourth-generation synchrotrons moving towards higher brilliance and flux densities. This is going to present an increasing problem that will surely require addressing, particularly as currently the SMX experiments conducted herein were done so under a heavily attenuated incident beam. Given that it is not possible to change the dose experienced by a sample, the parameter that should be explored is the sensitivity of a sample *i.e.* its propensity to decay given a particular dose.

Therefore, the next experiments to perform will be to compare the effects measured here at different synchrotron sources, given it will be possible to undertake systematic comparisons based on these results. Extending the studies herein would include investigating a broader range of sample types, which will enable different mechanisms for the observed decay to be explored. Unlike MX, which generally has similar ways for decay to be initiated and propagated across a range of different samples, it is possible to postulate a number of routes for this to occur in SMX. For example, it will be necessary to gain a better understanding of the extent to which different absorbing atomic centres affect decay and to use spectroscopic approaches to characterize the reaction of the sample decay. The effect of varying the incident X-ray energy should also be explored, but account will have to be taken of the varying efficiency of the X-ray detector with energy in such studies.

The goal of this scheme of work should be to understand different decay mechanisms with a view to developing the best approaches to mitigate them. Ultimately, this should lead to being able to anticipate the effect, design the optimal data-collection strategy for a given sample and experimental arrangement, and obtain the desired level of accuracy in the result. Furthermore, in order to develop such a complete understanding, it will be necessary to establish the error margins for the experiments conducted here, for example for crystal size. A potential route to explore for SMX is the merging of multiple lower-dose datasets – a procedure now well established in MX serial crystallography, but as yet essentially unexplored in SMX.

It is also very worthy and important to be mindful of the fact that the resolution and volume of SMX results is far greater than that of MX. Thus, for those researching radiation damage in MX, it is potentially valuable to study and learn about parameters such as unit-cell expansion or mosaicity from SMX, where models are defined much more precisely, and these findings could then be applied to the less well defined MX systems. 

## Supplementary Material

Crystal structure: contains datablock(s) I. DOI: 10.1107/S2052252519006948/lq5022sup1.cif

Structure factors: contains datablock(s) I. DOI: 10.1107/S2052252519006948/lq5022sup2.hkl

Supporting information - tables and figures. DOI: 10.1107/S2052252519006948/lq5022sup3.pdf

Experiment 1 dataset: https://doi.org/10.5281/zenodo.2592265

Experiment 2 dataset: https://doi.org/10.5281/zenodo.2551061

Experiment 3 dataset: https://doi.org/10.5281/zenodo.2552049

Experiment 4 dataset: https://doi.org/10.5281/zenodo.2553080

Experiment 5 dataset: https://doi.org/10.5281/zenodo.2556528

Experiment 6 dataset: https://doi.org/10.5281/zenodo.2557314

Experiment 7 dataset: https://doi.org/10.5281/zenodo.2587857

Experiment 8 dataset: https://doi.org/10.5281/zenodo.2591169

In-house benchmark dataset: https://doi.org/10.5281/zenodo.2591394

CCDC reference: 1901775

## Figures and Tables

**Table 1:** Indicative values for some experimental characteristics that are significantly different between MX and SMX where sample-decay effects might thus be expected to be markedly dissimilar

	MX	SMX
Water content	Nearly all samples contain 50–80% water	*ca* 10% of all samples are hydrates or contain 1–10 molecules of crystallization. (Threlfall, 1995 ▸; Infantes *et al.*, 2003 ▸)
Absorption cross-section at 8.05 keV (Cu *K*α) and 17.997 keV	Lysozyme, 1.04 and 0.080 mm^−1^; Insulin, 1.02 and 0.078 mm^−1^	Indicative absorption coefficient for this sample: 1.736 and 0.804 mm^−1^
Typical incident wavelength (Å)	1.5–0.9	1.5–0.4
Typical beam size	Large range of available sizes and often adjustable	Crystal smaller than beam
Typical crystal size	3–200 µm in any dimension	20–50 µm in all dimensions
Flux in incident beam (photons s^−1^)	10^12^	10^10^
Applicable no. of space groups	65	230
Multiplicity due to symmetry	1–4	1–10
Molecular weight (Da)	>20000	<900
Unit-cell size / number of reflections	>50 Å axes	<50 Å axes, <5000 unique reflections, <100000 reflections per dataset
Typical diffraction limit (Å)	3.5–1.5	Beyond 0.8
No. of scans on one crystal	1 (but depends on the phasing technique used)	3–6 depending on instrument geometry

**Table 2:** Overview of experiments conducted in order to probe the effect of the parameters thought to be most influential in affecting radiation-damage rates

Experiment number	Transmission (%)	Wavelength (Å)	Temperature (K)	Crystal size (µm)	Average DWD per scan (MGy)
1	1	0.6889	100	55 × 10 × 10	0.63
2	2	0.6889	100	50 × 10 × 10	1.27
3	0.5	0.6889	100	50 × 8 × 8	0.32
4	1	0.6889	100	25 × 10 × 5	0.57
5	1	0.9028	100	30 × 5 × 5	0.79
6	3	0.6889	30	50 × 8 × 6	1.90
7	3	0.6889	60	40 × 12 × 8	1.92
8	1	0.6889	120	50 × 10 × 10	0.60

**Table 3:** Dose and quality parameters for the three selected data points [see Fig. 6 ▸ for the definitions of (*a*), (*b*) and (*c*)]

Data point	Dose (MGy)	Resolution (Å)	*R*_merge_	*R*_1_	norm 〈*I*〉	〈*I*/σ(*I*)〉
(*a*)	1.26	0.65	0.13	0.0914	85.4	3.3
(*b*)	3.99	0.63	0.091	0.0932	57.0	5.1
(*c*)	3.78	0.77	0.173	0.1104	55.9	2.2
