# Supplementary material for: Radiation damage in small-molecule crystallography: fact not fiction
Source: IUCrJ. 2019 Jun 14;6(Pt 4):703–13. doi: 10.1107/S2052252519006948 (PMC6608633; doi:10.1107/S2052252519006948)
Supplement: Supplementary file 3 [file m-06-00703-sup3.pdf]

IUCrJ

Volume 6 (2019)

Supporting information for article:

Radiation damage in small-molecule crystallography: fact not fiction

Jeppe Christensen, Peter N. Horton, Charles S. Bury, Joshua L. Dickerson, Helena Taberman, Elspeth F. Garman and Simon J. Coles

S1. Beam characterisation

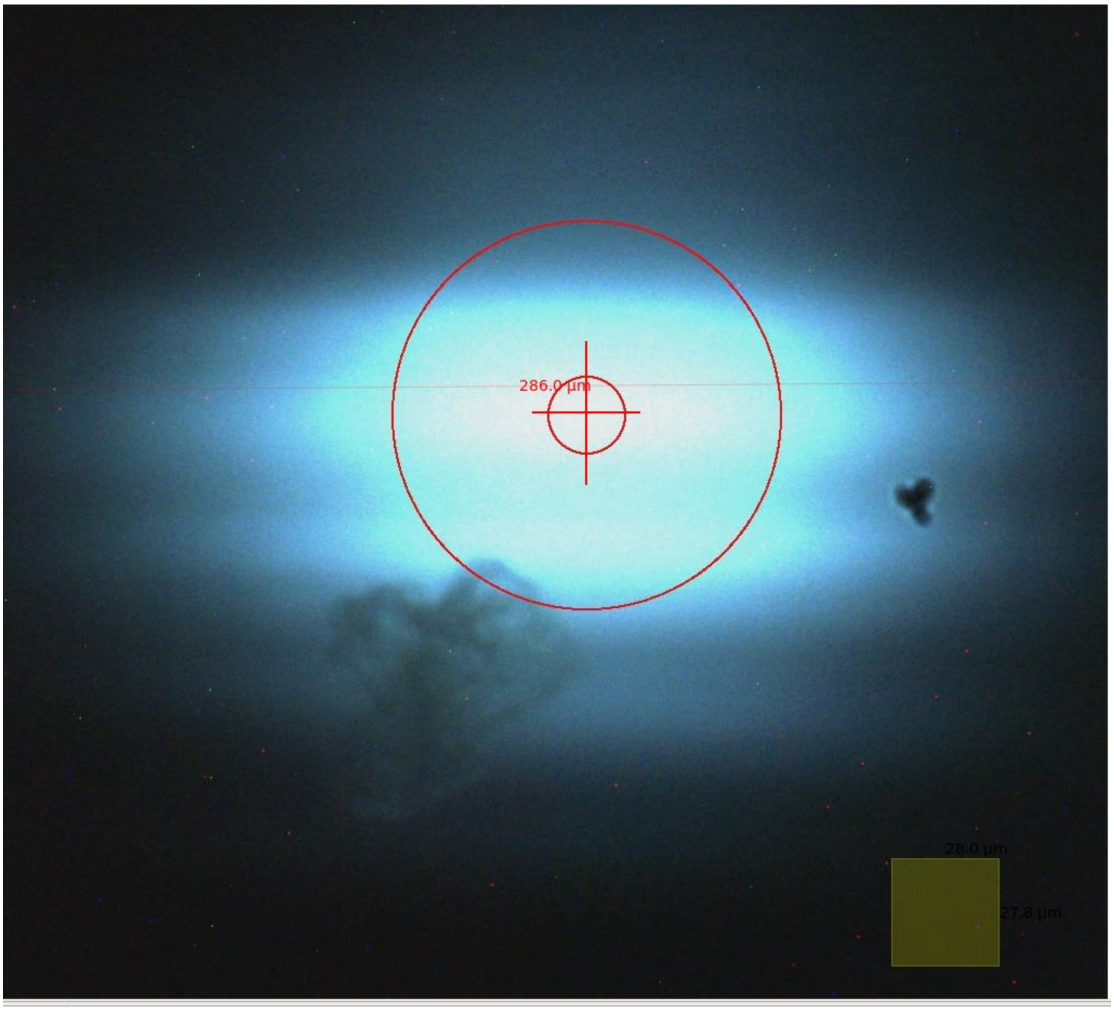

Figure S1 The X-ray beam on the sample position captured using a fluorescent screen and the on-axis viewing camera. The large circle represents 100 µm collimation.

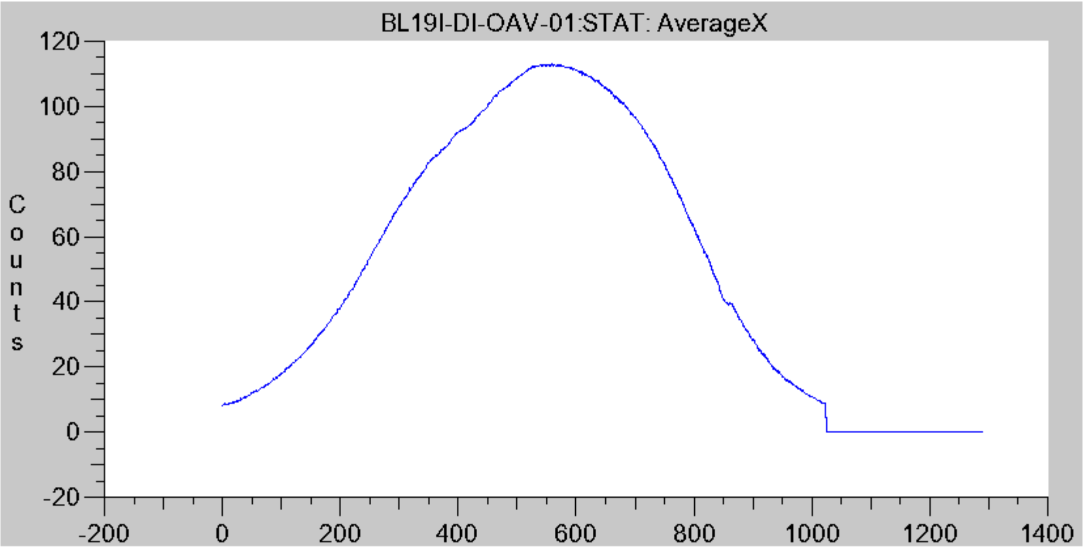

Figure S2 Horizontal beam profile measured from the image in figure S1. This was used to calculate the FWHM. The *x*-axis indicates the camera pixels (micrometres to pixels ratio 285:1024).

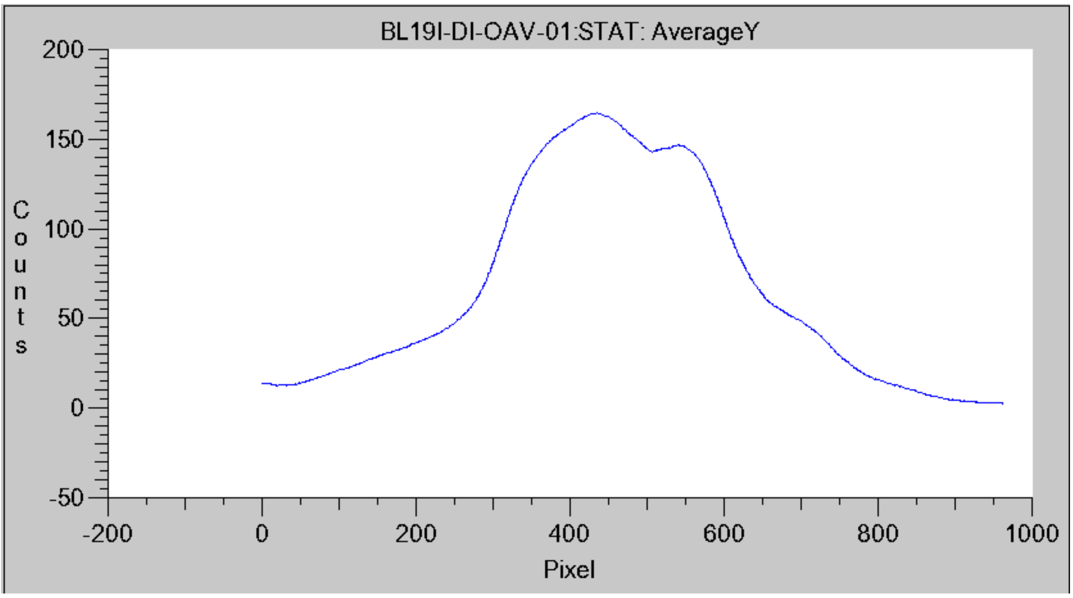

Figure S3 Vertical beam profile measured from the image in Figure S1. This was used to calculate the FWHM. The *x*-axis indicates the camera pixels (micrometres to pixels ratio 285:1024).

Table S1 Visit 1 at wavelength 0.6889 Å.

Transmission (%)	Diode reading (μA)	Flux/ph/s	Flux density ph/s/mm^2
100	339	8.78×10^{11}	1.12×10^{14}
1.97	6.69	1.73×10^{10}	2.20×10^{12}
1.00	3.39	8.8×10^{9}	1.12×10^{12}
0.50	1.68	4.35×10^{9}	5.54×10^{11}

Table S2 Visit 2 at wavelength 0.6889 Å.

Transmission (%)	Diode reading (μA)	Flux/ph/s	Flux density ph/s/mm^2
100	295	7.64×10^{11}	9.73×10^{13}
61.3	192	4.68×10^{11}	5.96×10^{13}
59.0	174	4.51×10^{11}	5.75×10^{13}
53.3	157	4.07×10^{11}	5.18×10^{13}
47.8	141	3.65×10^{11}	4.65×10^{13}
42.8	126	3.27×10^{11}	4.17×10^{13}
38.0	112	2.90×10^{11}	3.69×10^{13}
32.2	95.1	2.46×10^{11}	3.13×10^{13}
27.0	79.5	2.06×10^{11}	2.62×10^{13}
21.6	63.5	1.65×10^{11}	2.10×10^{13}
16.1	47.5	1.23×10^{11}	1.57×10^{13}
10.7	31.5	8.16×10^{10}	1.04×10^{13}
5.3	15.7	4.07×10^{10}	5.18×10^{12}
1.1	3.15	8.162×10^{9}	1.04×10^{12}

Table S3 Visit 3 at wavelength 0.9028 Å.

Transmission (%)	Diode reading (μA)	Flux/ph/s	Flux density ph/s/mm^2
100	490	1.036×10^{12}	1.32×10^{14}
24.7	121	2.558×10^{11}	3.26×10^{13}
5.47	26.8	5.665×10^{10}	7.22×10^{12}
1.22	5.99	1.266×10^{10}	1.61×10^{12}
0.64	3.125	$6.605\ \times10^{9}$	8.41×10^{11}
0.27	1.33	2.811×10^{9}	3.58×10^{11}
0.06	0.303	6.404×10^{8}	8.16×10^{10}

Table S4 Visit 4 at wavelength 0.6889 Å.

Transmission (%)	Diode reading (μA)	Flux/ph/s	Flux density ph/s/mm^2
100	318	8.239×10^{11}	1.05×10^{14}
65.4	208	5.389×10^{11}	6.86×10^{13}
55.0	175	4.534×10^{11}	5.78×10^{13}
44.0	140	3.627×10^{11}	4.62×10^{13}
32.7	104	2.625×10^{11}	3.34×10^{13}
21.8	69.5	1.80×10^{11}	2.29×10^{13}
10.7	34.1	8.835×10^{10}	1.13×10^{13}
1.1	3.38	8.757×10^{9}	1.12×10^{12}

Table S5 Visit 5 at wavelength 0.6889 Å.

Transmission (%)	Diode reading (μA)	Flux/ph/s	Flux density ph/s/mm^2
100	312	8.084×10^{11}	1.03×10^{14}
52.9	165	4.275×10^{11}	5.45×10^{13}
32.0	100	2.59×10^{11}	3.30×10^{13}
10.4	32.5	8.42×10^{10}	1.07×10^{13}
1.04	3.25	8.42×10^{9}	1.07×10^{12}

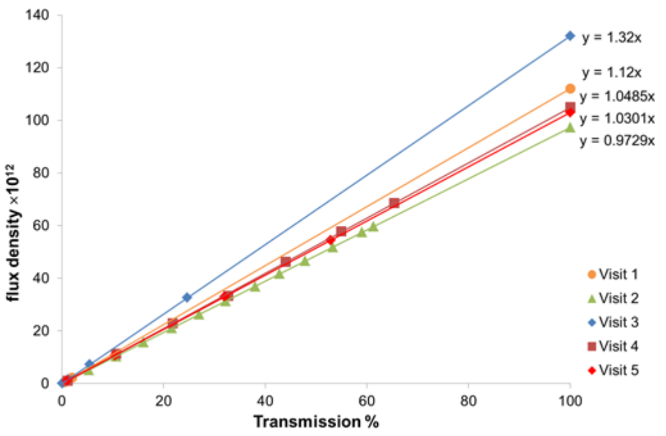

Figure S4 Comparison of calibration results from each visit. The significant visit-to-visit intensity variations illustrate the necessity to perform the calibration for each experiment.

S2. Experiment overview

Visit	Experiment	Transmission (%)	Wavelength (Å)	Temp (K)	Size (μm)	Average DWD per scan (MGy)	Link to repository record containing raw data and associated information
1	1	1	0.6889	100	55×10×10	0.63	https://doi.org/10.5281/zenodo.2592265
1	2	2	0.6889	100	50×10×10	1.27	https://doi.org/10.5281/zenodo.2551061
1	3	0.5	0.6889	100	50×8×8	0.32	https://doi.org/10.5281/zenodo.2552049
2	4	1	0.6889	100	25×10×5	0.57	https://doi.org/10.5281/zenodo.2553080
3	5	1	0.9028	100	30×5×5	0.79	https://doi.org/10.5281/zenodo.2556528
4	6	3	0.6889	30	50×8×6	1.90	https://doi.org/10.5281/zenodo.2557314
4	7	3	0.6889	60	40×12×8	1.92	https://doi.org/10.5281/zenodo.2587857
5	8	1	0.6889	120	50×10×10	0.60	https://doi.org/10.5281/zenodo.2591169
In-house benchmark			0.71073	100	70×10×10	Not calculated	https://doi.org/10.5281/zenodo.2591394

Table S6. Overview of the conditions for each experiment performed with a corresponding repository link to enable the download of the full raw data record.

For each experiment the raw diffraction images have been deposited in the Zenodo repository (https://zenodo.org/), with the specific data links to the corresponding record given in the table. The diffraction images are in CBF format, which is the community agreed exchange standard for diffraction data. It can be transformed into a number of other formats and read by processing software. The record also includes a CIF file containing the relevant beamline parameters, information about image format and suitable software for data processing.

S3. Results

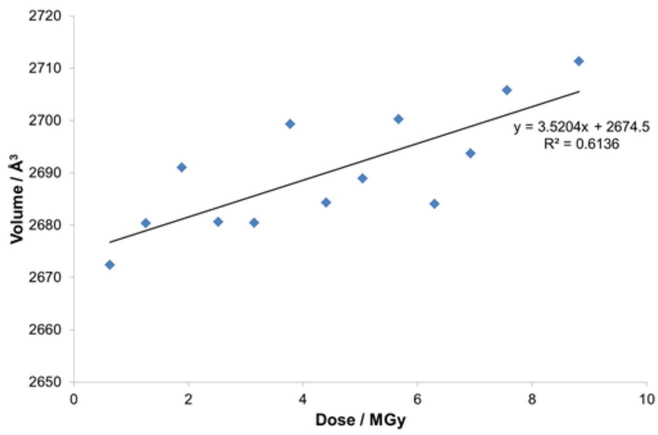

Figure S5 Unit cell expansion as a function of dose (average DWD) for Experiment 1.

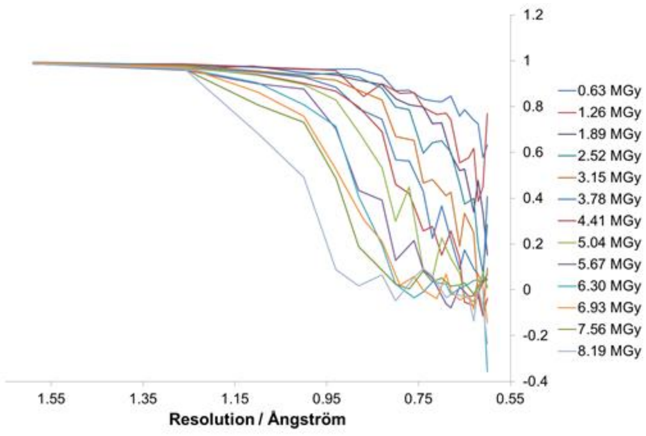

Figure S6 Plot indicating the value of $CC_{1/2}$ developing as a function of dose. The diffraction limit is calculated based on the point at which $CC_{1/2}$, which is a correlation coefficient for anomalous differences and equivalent mean intensities, <I>, between two halves of a randomly split dataset, falls below a threshold (the default value used in the XIA2 software used for this work is 0.3).

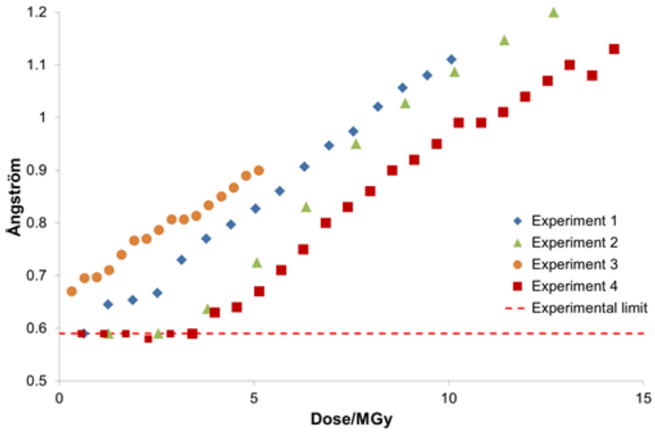

Figure S7. The effect of beam intensity on resolution decay rate. Experiments 1 and 4 were performed at 1% beam transmission, Experiment 2 at 2% beam and Experiment 3 at 0.5% beam transmission.

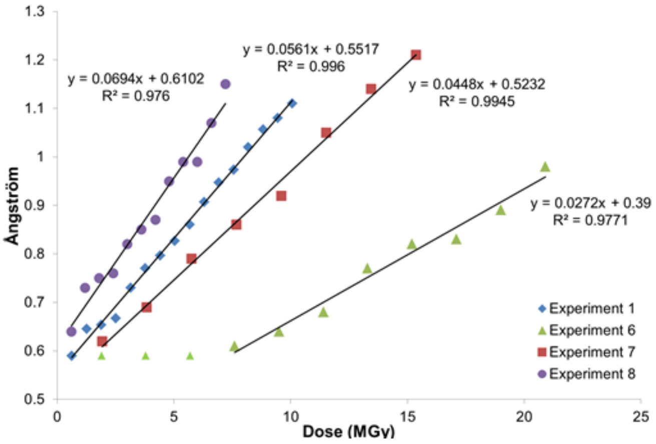

Figure S8 The effect of temperature on sample decay rate. Experiment 8 was performed at 120K, Experiment 1 at 100K, Experiment 7 at 60K and Experiment 6 at 30K.

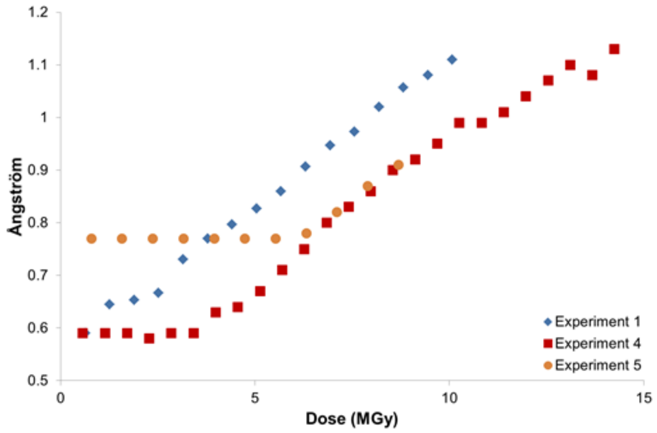

Figure S9 The effect of incident beam energy (wavelength). Experiments 1 and 4 were performed at 0.6889 Å and Experiment 5 at 0.9028 Å. The detector angular acceptance limits the initial resolution therefore differs for the two wavelengths**.**
